# ABHD5 as a friend or an enemy in cancer biology?

**DOI:** 10.3389/fonc.2024.1447509

**Published:** 2024-09-12

**Authors:** Jianya Cai, Hongwei Cheng, Shuangta Xu

**Affiliations:** ^1^ Department of Surgery, Quanzhou Medical College, Quanzhou, China; ^2^ Zhuhai UM Science & Technology Research Institute, University of Macau, Macau, Macau SAR, China; ^3^ Department of Thyroid and Breast Surgery, The Second Affiliated Hospital of Fujian Medical University, Quanzhou, China

**Keywords:** ABHD5, lipid metabolism, proliferation, invasion, oncogene

## Abstract

Alpha beta hydrolase domain containing 5 (ABHD5) is an essential coactivator of adipose triglyceride lipase (ATGL), a rate-limiting enzyme in various cell types that promotes the hydrolysis of triacylglycerol (TG) into diacylglycerol (DG) and fatty acid (FA). It acts as a critical regulatory factor in cellular lipid metabolism. The reprogramming of lipid metabolism is one of the hallmarks of cancer, suggesting that altering lipid metabolism could become a new strategy for tumor treatment. Research has revealed a close association between ABHD5 and the development and progression of malignancies. This review summarizes the role of ABHD5 in various malignant tumors and explores the different signaling pathways and metabolic routes that may be involved, providing a comprehensive mechanistic understanding of ABHD5.

## 1 Introduction

With the rapid development of molecular biology, an increasing number of molecular factors have been discovered to participate in the complex processes of cancer initiation, proliferation, invasion, and metastasis. Among these factors, the dysregulation of lipid metabolism has attracted significant attention from the global scientific community (1–3). Abnormal lipid metabolism not only affects the structure and function of cell membranes but also directly impacts crucial physiological processes such as cell signaling, metabolic pathway regulation, and energy balance (4).

In the study of lipid metabolism, the α/β hydrolase domain (ABHD) family has garnered considerable attention because of its important role (5). The ABHD family belongs to the α/β hydrolase superfamily and is characterized by a distinctive α/β hydrolase fold structure. Members of this family contain eight β-strands, with the second β-strand being antiparallel. These β-strands are flanked on both sides by α-helices and loops that connect the eight β-strands (5). Most members of the ABHD family possess a typical esterase catalytic triad, which usually comprises an acidic residue (glutamate or aspartate), a histidine, and a nucleophile. Members of the ABHD family perform a variety of biological functions, including regulating lipid metabolism, signal transduction, and energy balance. Through various enzymatic activities and regulatory mechanisms, they influence the synthesis, breakdown, and remodeling of lipids within cells (6). Several members of the ABHD family have been implicated in the development and progression of malignancies. For example, ABHD2 has been associated with the invasiveness of breast cancer cells (7), ABHD3 was identified in a pro-apoptotic gene screen (8), ABHD6 promotes colorectal cancer progression (9), but the tumor-suppressive effects have also been reported on non-small cell lung cancer (10), and ABHD9 is linked to prostate cancer recurrence (11, 12).

ABHD5, also known as comparative gene identification-58 (CGI-58), stands out as a key member of the ABHD family and plays a vital role in cellular triglyceride metabolism. By activating adipose triglyceride lipase (ATGL), ABHD5 promotes the hydrolysis of triglycerides, thereby regulating intracellular lipid homeostasis (13). In various types of cancer, ABHD5 is abnormally expressed and is involved in the proliferation, invasion, and metastasis of tumor cells. This review summarizes the dual roles of ABHD5 in cancer, either as a tumor suppressor or promoter, and explores the potential of ABHD5 as a therapeutic target for cancer.

## 2 Discovery and basic structure of ABHD5

**Figure 1:**
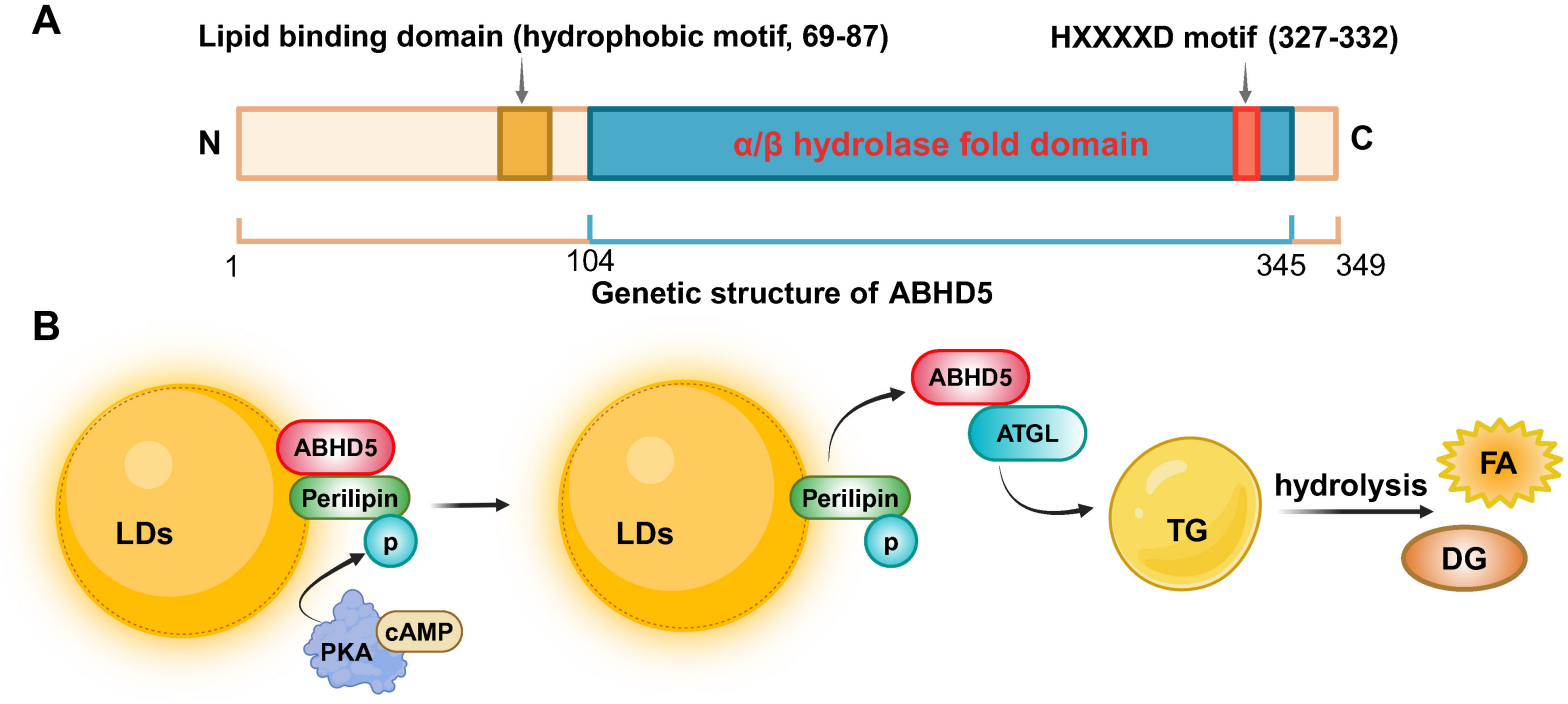
The genetic structure of ABHD5 and its regulatory mechanism in lipid metabolism. **(A)** The ABHD5 gene is encoded by 349 amino acids and consists of an N-terminal lipid binding domain, an α/β hydrolase fold domain, and a C-terminal HXXXXD motif with acyltransferase activity. **(B)** The mechanism signaling about ABHD5 in the regulation of lipid metabolism. Created by BioRender.com.

ABHD5 was initially identified in relation to neutral lipid storage disease with ichthyosis (NLSDI) (14, 15)or chanarin-Dorfman syndrome (CDS) (16, 17). These patients exhibit severe ichthyosis, characterized by dry and scaly skin, which is a result of disrupted lipid storage and metabolism. This syndrome exemplifies the complex interplay of metabolic anomalies, with lipid-laden cytoplasmic droplets accumulating not only in the skin’s epidermal layers but also in various cell types across the body, including leukocytes, hepatocytes, skeletal muscle, and even cells within the central nervous and auditory systems. This mutation leads to triacylglycerol (TG) accumulation across various nonadipose tissues, illustrating ABHD5’s critical role in regulating cellular lipid homeostasis. The α/β-hydrolase fold superfamily, to which ABHD5 belongs, is one of the most widely distributed and functionally diverse protease superfamilies in nature. Characterized by the α/β hydrolase fold domain, the structural size of this superfamily ranges from 197 to 583 amino acids, with molecular weights varying from 25 to 65 kDa (18). ABHD5, encoded by the CGI-58 gene located on chromosome 7p21.33, consists of 8 exons and produces a 349-amino acid protein. Its unique α/β-hydrolase fold, comprising eight α-helices and β-sheets, allows it to interact with a variety of substrates (19). Although ABHD5 contains a sequence common among members of the esterase/lipase/thioesterase subfamily, its active serine residue in the GXSXG motif is replaced by aspartic acid, resulting in the absence of inherent lipase activity. In addition, the carboxyl terminus of ABHD5 contains a highly conserved consensus sequence (HXXXXD) motif that has acyltransferase activity, whereas the N-terminus contains a hydrophobic motif that is also a lipid-binding domain (**Figure 1A**). Despite lacking esterase activity itself, ABHD5 can interact with ATGL, activating its enzymatic activity and playing a crucial regulatory role in lipolysis (5).

## 3 Roles of ABHD5 in cellular metabolism

ABHD5 is a widely expressed protein that is highly expressed in tissues such as adipose tissue, testes, liver, kidneys, heart, and skin (20–22). It plays a crucial role in regulating lipid metabolism within cellular metabolic processes. ABHD5 interacts with ATGL to facilitate the hydrolysis of TG (20). Specifically, in resting adipocytes, ABHD5 colocalizes with perilipin on the surface of lipid droplets (LDs). Upon certain hormonal signals, such as an increase in cyclic adenosine monophosphate (cAMP), protein kinase A (PKA) is activated, triggering the dissociation of ABHD5 from perilipin (**Figure 1B**). ABHD5 is then rapidly released from LDs into the cytoplasm, promoting ATGL activity and catalyzing the hydrolysis of TG into diacylglycerol (DG) and fatty acid (FA). These fatty acids can be further utilized by the cell for energy production or other metabolic pathways (21, 23, 24). When Plin5 is in an unphosphorylated state, it can bind to ABHD5, limiting its activity and thus inhibiting ATGL activation (25, 26). Additionally, ABHD5 is involved in the secretion of lipoproteins. In hepatic cells, after triacylglycerols are packaged into lipoprotein particles, ABHD5 aids in the transport of these particles to the extracellular space and their release into the bloodstream (27).

## 4 Dual roles of ABHD5 in cancer

Metabolic reprogramming is a hallmark of cancer and involves enhanced aerobic glycolysis, altered glutamine metabolism, and adjusted lipid handling to meet the energy and material needs of rapidly growing tumor cells (28–30). Additionally, metabolic reprogramming helps tumor cells adapt to hypoxic and nutrient-deprived microenvironments, supporting their growth and survival (31). As a key regulator of lipid metabolism, ABHD5 plays a significant role in tumor metabolic reprogramming, particularly in the regulation of lipid metabolism. By controlling triglyceride metabolism, ABHD5 influences the synthesis and breakdown of fatty acids, thereby affecting the energy metabolism of tumor cells (20). There are considerable differences among various malignant tumors, such as differences in the microenvironment, metabolic demands, interactions among different signaling pathways, and cellular responses to ABHD5. Consequently, ABHD5 plays dual roles, acting either as a tumor suppressor or promoter in different cancers, as detailed in **Table 1**.

**Table 1:** The expression of ABHD5 and related signaling in some cancers.

Cancer	Tissue	Cell line	Expression	Pathway	Function	Ref
Colorectal cancer	–	HCT116 SW620	–	YAP/cMet (Hippo)	Suppress cancer cell stemness by promoting YAP activity and nuclear localization through SET1A-mediated YAP methylation	(32)
	Tumor	–	Low	PI3K/Akt/mTOR/p53	The absence of ABHD5 directly inhibits the AMPK-p53 pathway, thereby promoting the occurrence of EMT	(33)
	Paracancerous		High			
	–	HCT16	–			
	Tumor	–	–	ROS-inflammasome pathway	Colon cancer with low ABHD5 expression is more progressed. Autophagy uracil regulation by ABHD5 increases colorectal cancer FU sensitivity	(34)
		SW480 FET	–			
	Tumor	–	–	Autophagy	ABHD5 competes with CASP3 to bind to the BECN1 cleavage site, blocking cleavage and encouraging tumor development via autophagy	(35)
	–	CCD841CON FHC	–			
	Tumor	–	–	NF-κB/MMP	Macrophage ABHD5 reduces tumor metastasis by inhibiting the expression of NF-κB-dependent MMPs.	(36)
	–	CTR-26 MC-38	–			
	–	RAW264.7, BMDMs PMs	–			
	Tumor	–	–	C/EBP_Ɛ_/SRM	TAMs’ ABHD5 inhibits ROS-dependent C/EBP_Ɛ_ expression to reduce SRM-dependent arginine synthesis, boosting SRM gene transcription and promoting tumor proliferation	(37)
	–	HCT116 SW480 MC-38	–			
		RAW	–			
Liver Cancer	–	McA-RH7777	–	–	ABHD5 promotes the secretion of lipoproteins in liver cancer	(27)
		HepG2				
	Tumor	–	Low		Downregulation of ABHD5 leads to increased expression of PD-L1 in liver cancer	(38)
	Paracancerous		High			
	–	SMMC7721 SK-Hep-1		PD-L1		
Lung cancer	Tumor	–	Low	NF-κB-pathway	Suppress the proliferation, migration, and invasion and induce apoptosis	(39)
	Paracancerous		High			
	–	A549 HCC827	–			
Prostatic cancer	–	LNCaP C4-2 C4-2B	–	–	Inhibit the invasion and proliferation	(40)
	–	C4-2 22RVl	–	PI3K/Akt/mTOR	Inhibit the anabolism of cancer cells by activating the AMPK/mTORC1 pathway	(41)
	–	LNCaP HeLa OP9	–	Phosphorylation by P70S6 kinase	Knockdown of ABHD5 can reduce the phosphorylation of P70S6 protein, leading to induction of tumor apoptosis	(42)
Endometrial cancer	Tumor	–	High	AKT signaling pathway	ABHD5 could promote cell proliferation in endometrial cancer cells through the AKT signaling pathway	(43)
	Paracancerous		Low			
	–	HEC-1A Ishikawa RL952 AN3CA	–			
	Tumor	–	High	–	Overexpression of ABHD5 acts a poor prognostic marker	(44)
	Paracancerous		Low			

### 4.1 Colorectal cancer

ABHD5 has been validated as a tumor suppressor gene in colorectal cancer by multiple laboratories. Gu highlighted the role of ABHD5 in maintaining the properties of colorectal cancer stem cells (32). Specifically, ABHD5 interacts with the SET1A methyltransferase complex, inhibiting the nuclear localization of DPY30 and the activity of SET1A. Without ABHD5, DPY30 can enter the nucleus, where it promotes the methylation of YAP and histone H3, thereby activating the transcription of c-Met and supporting stem cell survival in colorectal cancer (CRC).

In Ou’s research, the inhibitory role of ABHD5 in the epithelial−mesenchymal transition (EMT) of colorectal cancer cells was explored (33). EMT is a crucial process in cancer metastasis, and the absence of ABHD5 expression has been demonstrated to increase the invasiveness of HCT116 cells and increase tumor growth in the lungs of a nude mouse model. ABHD5 deficiency directly suppresses the AMPK−p53 pathway, thereby promoting EMT. In the Apc^Min/+^ mouse model, specific deletion of ABHD5 led to an increase in the number and size of tumors in the colon and rectum, indicating that ABHD5 plays a significant role in inhibiting the development of colorectal cancer. Furthermore, the level of ABHD5 expression in human CRC tissues is inversely correlated with the degree of CRC malignancy, suggesting that ABHD5 may influence the malignant transformation of cancer by regulating metabolic effects such as the Warburg effect. Therefore, defects in ABHD5 may be associated with the progression of colorectal cancer and its transformation from adenoma to carcinoma.

The research team subsequently explored the role of ABHD5 in the sensitivity of colorectal cancer cells to 5-fluorouracil (5-FU)-based chemotherapy (34). ABHD5 plays a pivotal role in enhancing the responsiveness of colorectal cancer to 5-FU by regulating autophagy-related uracil production. Experiments have demonstrated that ABHD5 interacts with PDIA5 in lysosomes to prevent the inactivation of RNASET2 by PDIA5. In the absence of ABHD5, this interaction is hindered, affecting autophagy-related uracil production and thus increasing the sensitivity of colorectal cancer cells to 5-FU. This article also discusses the significance of ABHD5 status in predicting the benefits of 5-FU-based adjuvant chemotherapy in patients with proficient mismatch repair (pMMR). These findings reveal the relationship between ABHD5 and the susceptibility of colorectal cancer cells to 5-FU, providing a potential strategy to overcome chemoresistance in the treatment of colorectal cancer.

Peng’s study explored how the interaction between ABHD5 and BECN1 regulates autophagy and affects the development of colorectal cancer (35). Research has indicated that ABHD5 directly competes with CASP3 at the cleavage site of BECN1, thereby preventing its cleavage and influencing the autophagy pathway, which may contribute to tumorigenesis. In addition to its role in activating PNPLA2 to promote triglyceride breakdown, ABHD5 independently regulates the autophagic process. Moreover, the absence of ABHD5 may exacerbate DNA damage. Cells lacking ABHD5 exhibit increased sensitivity to genotoxic damage and chromosomal instability, which can be mitigated by treatment with the mTOR inhibitor rapamycin. Clinical data further confirmed a significant correlation between the expression of ABHD5 and the expression of BECN1, LC3-II, and CASP3 in human colorectal cancer tissues. These findings underscore the role of ABHD5 as a tumor suppressor and suggest potential avenues for developing new strategies to prevent colorectal cancer.

The role of ABHD5, a lipid metabolism regulator, in metabolic reprogramming in tumor-associated macrophages (TAMs) was reported by Shang’s group (36). Research groups have reported heterogeneous expression of the lipolytic coactivator ABHD5 in TAMs, with migratory TAMs demonstrating lower levels of ABHD5 than their nonmigratory counterparts. Investigations conducted *in vitro*, in xenograft models, and in genetic cancer models have demonstrated that ABHD5 expression in macrophages suppresses the migration of cancer cells. This suppressive effect of macrophage ABHD5 on cancer cell migration is independent of its metabolic functions, as neither triglycerides nor metabolites regulated by ABHD5 impact cancer cell migration. Instead, ABHD5 exerts its influence through another mechanism, specifically by inhibiting the expression of NF-κB-dependent matrix metalloproteinases (MMPs). A negative correlation was observed between ABHD5 expression in TAMs and MMP expression. In colorectal cancer patients, higher levels of ABHD5 are associated with better survival rates. Overall, this study indicates that ABHD5 in macrophages inhibits the production of MMPs and cancer metastasis, making ABHD5 a prognostic marker for CRC.

In a follow-up study, Miao’s team intriguingly discovered an increase in the expression of ABHD5 within TAMs in colorectal cancer. *In vitro* and in mouse models, macrophage ABHD5 suppressed the expression of C/EBPe in response to reactive oxygen species, inhibiting the generation of spermidine via spermine synthase (SRM), thereby promoting the proliferation of colorectal carcinoma cells (37). These findings suggest that TAMs could be therapeutically targeted by targeting the ABHD5/SRM/spermidine axis. This research not only offers a new perspective on the role of TAMs in colorectal cancer but also reveals the ABHD5/SRM/polyamine pathway as a potential therapeutic target, opening avenues for novel treatment strategies. These results contradict the conclusions drawn by Shang et al., suggesting that the role of ABHD5 in cancer is quite complex, with different studies reporting divergent outcomes concerning whether ABHD5 acts as an oncogene or a tumor suppressor. In summary, the contrasting outcomes regarding ABHD5 in colorectal cancer cells and TAMs suggest that its mechanism of action is subject to multifaceted regulation, which varies depending on specific cellular contexts or environmental conditions. Studies indicate that ABHD5 can exhibit both tumor-suppressive and tumor-promoting effects, underscoring its complex role in the TME.

### 4.2 Liver cancer

Brown’s research delves into the role of ABHD5 in hepatocellular lipid metabolism. This study revealed that ABHD5 promotes the encapsulation of cytoplasmic TG into secretory lipoprotein particles in liver tumor cells. Using gain- and loss-of-function methods, ABHD5 was shown to increase the utilization of cellular TG stores without affecting the accumulation of cholesterol or phospholipids. This process is attributed entirely to increased TG hydrolysis, with TG synthesis not being affected by ABHD5. Additionally, ABHD5-mediated TG hydrolysis is associated with increased fatty acid oxidation and increased TG secretion. These findings establish a vital function of ABHD5 in the degradation, oxidation, and packaging of cytoplasmic TG into lipoprotein particles that transport TG out of liver cancer cells to diverse tissues. These findings help elucidate the cellular destiny of TG and the implications of ABHD5 functional loss, which leads to the abnormal hepatic lipid accumulation observed in conditions such as CDS (27).

Xu discovered that in hepatocellular carcinoma (HCC) tissues, the expression of ABHD5 is significantly downregulated, and this downregulation is notably correlated with poor prognosis in liver cancer patients, suggesting that ABHD5 is a novel therapeutic target. Research has also investigated the potential relationship between ABHD5 and tumor immune checkpoint molecules, particularly its interactions with programmed death-ligand 1 (PD-L1). PD-L1 is a well-established target of immune checkpoint blockade (ICB) therapy; therefore, understanding this interaction mechanism is crucial for enhancing the treatment efficacy of HCC. This study paves the way for new treatment possibilities by shedding light on the dual role of ABHD5 in immune regulation—as a tumor-promoting factor and a potential immunotherapeutic target. Future research may delve deeper into the specific mechanisms of ABHD5 within the tumor immune microenvironment and assess its potential as both a prognostic marker and a predictive indicator of response to ICB therapy (38).

### 4.3 Lung cancer

Zhou’s research highlights the role of ABHD5 in lung adenocarcinoma. circ_cMras, ABHD5 and ATGL were expressed at low levels in lung adenocarcinoma cells and tissues. Circ_cMras suppressed lung adenocarcinoma cell progression via the NF-κB pathway by regulating the ABHD5/ATGL axis. As a downstream target of circ_cMras, ABHD5 may function as an antitumor agent to inhibit lung cancer progression (39).

### 4.4 Prostate cancer

Chen’s research revealed the role of ABHD5 as a co-factor for lipase, which plays a role in the behavioral patterns and invasiveness of prostate cancer (PCa) cells. Specifically, ABHD5 expression is significantly reduced in metastatic castration-resistant prostate cancer (mCRPC) and is inversely correlated with cell invasiveness. Lower ABHD5 levels are associated with greater invasiveness and intracellular TG levels. Knocking down ABHD5 in LNCaP cells promotes epithelial−mesenchymal transition (EMT), enhancing cancer cell mobility and invasiveness. These findings suggest that ABHD5 dysregulation plays a critical role in prostate cancer progression, highlighting its potential as a therapeutic target (40).

Chen et al. reported in another study that ABHD5 acts as a tumor suppressor in four types of cancers, namely, lung cancer, gastric cancer, liver cancer, and ovarian cancer, on the basis of data from the public database Kaplan−Meier Plotter. Patients with high ABHD5 expression presented significantly better survival curves than did those with low ABHD5 expression. This study demonstrated that the expression of ABHD5 reduced the proliferation rate of 22Rv1-ABHD5 and C4-2-ABHD5 cells and decreased the proportion of cells in the S phase. ABHD5 also facilitates the hydrolysis and resynthesis of TG by activating them. This cycle activates AMPK and subsequently inhibits mTORC1, thereby suppressing cancer cell synthetic metabolism (41).

A study by Mitra revealed that prostate cancer cells overexpress ABHD5. Knocking down ABHD5 can promote lipid droplet accumulation and induce death in prostate cancer cells by triggering apoptosis through mechanisms involving the AMPK/P70S6 pathway. Additionally, this study revealed that both diacylglycerol acyltransferase-1 (DGAT1) and ABHD5 are upregulated in prostate cancer cells, suggesting that inhibiting these two proteins could be novel anticancer approaches. However, owing to the potential systemic side effects and lethality of ABHD5 inhibition (ABHD5 gene knockout in animal models is lethal, and mice lacking ABHD5 die within hours of birth), no inhibitors targeting ABHD5 are currently in development. This study suggests that targeting ABHD5 requires a deeper understanding of its functions beyond its interaction with ATGL, limiting the current application of a dual inhibition strategy targeting both ABHD5 and DGAT1 (42). The findings regarding the role of ABHD5 in prostate cancer differ between the two research teams. These opposing conclusions suggest that ABHD5 may influence tumor development through multiple distinct mechanisms.

### 4.5 Endometrial cancer

Zhou’s study revealed that ABHD5 is overexpressed in endometrial cancer and is linked to International Federation of Gynecology and Obstetrics (FIGO) stage and lymph node metastasis. High ABHD5 expression is correlated with poorer survival rates, suggesting that it is an adverse prognostic indicator. Downregulation of ABHD5 significantly reduces HEC-1A cell growth, tumor volume, and weight in both *in vitro* and *in vivo* models. ABHD5 promotes cell proliferation and invasion by regulating EMT and enhancing the Warburg effect (i.e., aerobic glycolysis enhancement) through the AKT signaling pathway and reduces HEC-1A cell growth, tumor volume, and weight in both *in vitro* and *in vivo* models (43). Shi, from the same research team as Zhou, reported that high ABHD5 expression is associated with poor overall survival (OS) in endometrial cancer patients, as confirmed by univariate and multivariate Cox regression analyses. ABHD5 may serve as a potential biomarker for endometrial cancer (44).

### 4.6 Others

Senchenko et al. identified a high frequency of ABHD5 methylation/deletion in cervical cancer using NotI microarray analysis of 48 paired samples. This finding supports the tumor-suppressive role of ABHD5 (45). Chen’s study analyzed 51 clinical samples, including 23 sebaceous carcinomas, 14 sebaceous adenomas, and 14 basal cell carcinomas with clear cell features. Immunohistochemical analysis revealed high ABHD5 expression in sebaceous tumors, suggesting ABHD5 as a potential diagnostic marker for sebaceous carcinoma (46). Similarly, the findings by Plaza et al. supported Chen’s results. Their study examined 27 sebaceous carcinoma cases, 21 basal cell carcinoma cases, and 22 squamous cell carcinoma cases, with extensive immunohistochemical testing validating the diagnostic significance of ABHD5 in sebaceous carcinoma (47).

## 5 Perspective and conclusion

**Figure 2:**
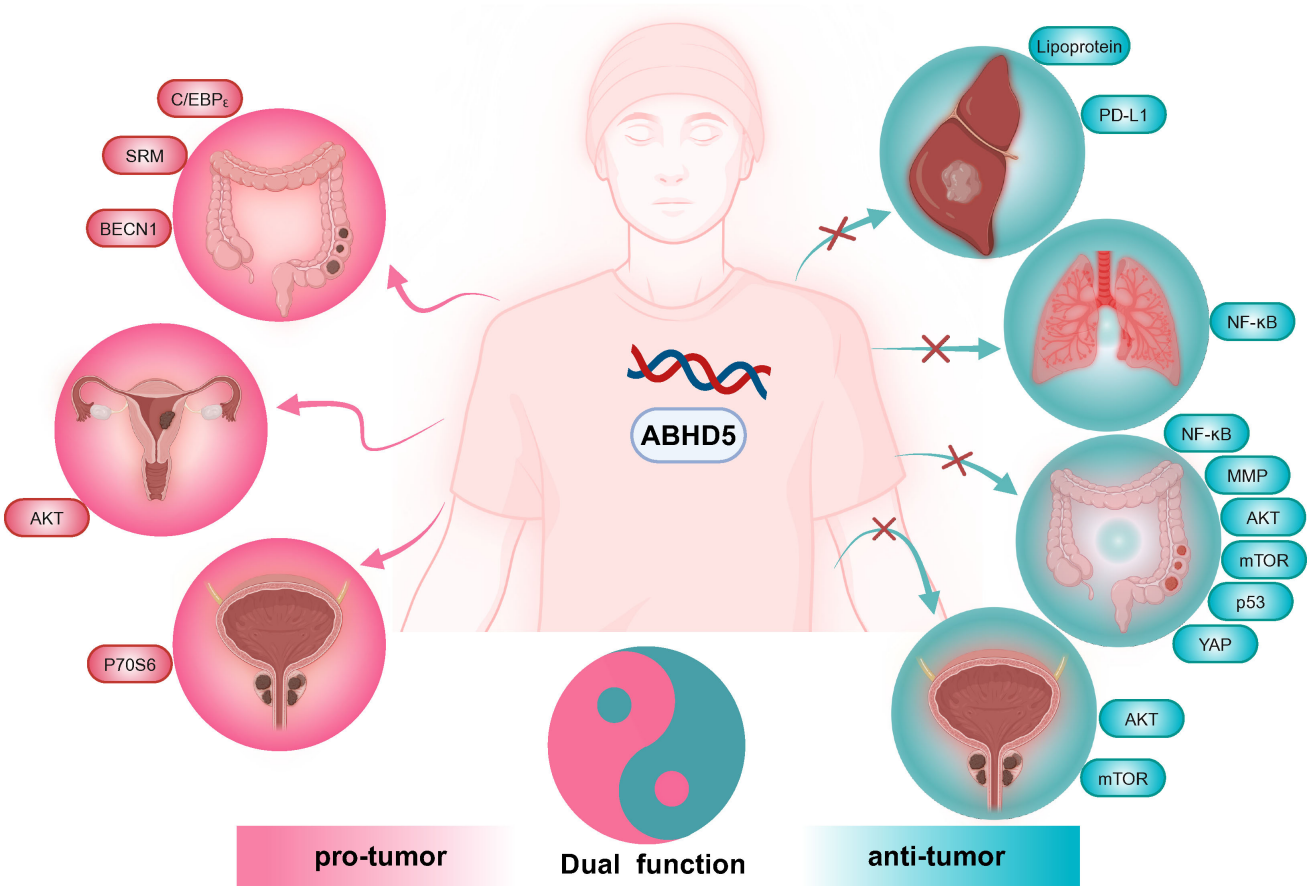
Effects of ABHD5 on carcinogenesis and anticarcinogenesis. In liver and lung cancer, ABHD5 functions as a tumor suppressor. Conversely, in endometrial cancer, ABHD5 acts as an oncogenic molecule. In prostate and colorectal cancers, ABHD5 plays dual roles.

ABHD5 is a protein involved in lipid metabolism that has recently garnered significant attention in oncology research due to its diverse roles across various tumor types. Studies have shown that ABHD5 can exhibit either oncogenic or tumor-suppressive effects depending on the context (**Figure 2**). This complexity suggests that the function of ABHD5 in tumorigenesis is influenced by multiple factors, including cell type and microenvironment. By investigating in detail how ABHD5 operates within different cellular subtypes and microenvironments, we can gain a deeper understanding of its multifaceted role in tumor progression. Such insights will not only help elucidate the mechanisms of cancer development but also guide the development of novel therapeutic strategies. Additionally, considering the impact of genetic background and patient-specific differences on ABHD5 function will lay the foundation for advancements in personalized medicine, providing new diagnostic methods and more precise treatment options for patients.

In conclusion, ABHD5 cannot be simply categorized as either an oncogene or a tumor suppressor, as its role is highly context-dependent and influenced by various factors. Comprehensive research into the mechanisms and regulatory networks involving ABHD5 in malignancies will not only enhance our understanding of its complex role in cancer progression but also drive the exploration and development of innovative cancer therapies.

## References

[1] Wang YY Lehuédé C Laurent V Dirat B Dauvillier S Bochet L. Adipose tissue and breast epithelial cells: a dangerous dynamic duo in breast cancer. Cancer Lett. (2012) 324:142–51. doi: 10.1016/j.canlet.2012.05.019 22643115

[2] Yao CH Fowle-Grider R Mahieu NG Liu GY Chen YJ Wang R. Exogenous fatty acids are the preferred source of membrane lipids in proliferating fibroblasts. Cell Chem Biol. (2016) 23:483–93. doi: 10.1016/j.chembiol.2016.03.007 PMC5510604 27049668

[3] Corn KC Windham MA Rafat M. Lipids in the tumor microenvironment: From cancer progression to treatment. Prog Lipid Res. (2020) 80:101055. doi: 10.1016/j.plipres.2020.101055 32791170 PMC7674189

[4] Cheng C Geng F Cheng X Guo D. Lipid metabolism reprogramming and its potential targets in cancer. Cancer Commun (Lond). (2018) 38:27. doi: 10.1186/s40880-018-0301-4 29784041 PMC5993136

[5] Bononi G Tuccinardi T Rizzolio F Granchi C. [amp]]alpha;/β-hydrolase domain (ABHD) inhibitors as new potential therapeutic options against lipid-related diseases. J Med Chem. (2021) 64:9759–85. doi: 10.1021/acs.jmedchem.1c00624 PMC8389839 34213320

[6] Xu J Gu W Ji K Xu Z Zhu H Zheng W. Sequence analysis and structure prediction of ABHD16A and the roles of the ABHD family members in human disease. Open Biol. (2018) 8:180017. doi: 10.1098/rsob.180017 29794032 PMC5990648

[7] Cao XH Chen X Yang K Wang YL Liang MX Fei YJ. Vaspin accelerates the proliferation, invasion and metastasis of Triple-Negative breast cancer through MiR-33a-5p/ABHD2. Cancer Med. (2023) 12:4530–42. doi: 10.1002/cam4.5241 PMC9972114 36125462

[8] Lin B Huntley D Abuali G Langley SR Sindelar G Petretto E. Determining signalling nodes for apoptosis by a genetic high-throughput screen. PLoS One. (2011) 6:e25023. doi: 10.1371/journal.pone.0025023 21966401 PMC3178610

[9] Xiong X Yang C Jin Y Zhang R Wang S Gan L. ABHD6 suppresses colorectal cancer progression via AKT signaling pathway. Mol Carcinog. (2024) 63:647–62. doi: 10.1002/mc.23678 38197491

[10] Tang Z Xie H Heier C Huang J Zheng Q Eichmann TO. Enhanced monoacylglycerol lipolysis by ABHD6 promotes NSCLC pathogenesis. EBioMedicine. (2020) 53:102696. doi: 10.1016/j.ebiom.2020.102696 32143183 PMC7057193

[11] Weiss G Cottrell S Distler J Schatz P Kristiansen G Ittmann M. DNA methylation of the PITX2 gene promoter region is a strong independent prognostic marker of biochemical recurrence in patients with prostate cancer after radical prostatectomy. J Urol. (2009) 181:1678–85. doi: 10.1016/j.juro.2008.11.120 19233404

[12] Cottrell S Jung K Kristiansen G Eltze E Semjonow A Ittmann M. Discovery and validation of 3 novel DNA methylation markers of prostate cancer prognosis. J Urol. (2007) 177:1753–8. doi: 10.1016/j.juro.2007.01.010 17437806

[13] Schratter M Lass A Radner FPW. ABHD5-A regulator of lipid metabolism essential for diverse cellular functions. Metabolites. (2022) 12:1015. doi: 10.3390/metabo12111015 36355098 PMC9694394

[14] Chanarin I Patel A Slavin G Wills EJ Andrews TM Stewart G. Neutral-lipid storage disease: a new disorder of lipid metabolism. Br Med J. (1975) 1:553–5. doi: 10.1136/bmj.1.5957.553 PMC1672681 1139147

[15] Slavin G Wills EJ Richmond JE Chanarin I Andrews T Stewart G. Morphological features in a neutral lipid storage disease. J Clin Pathol. (1975) 28:701–10. doi: 10.1136/jcp.28.9.701 PMC475811 1165295

[16] Missaglia S Coleman RA Mordente A Tavian D. Neutral lipid storage diseases as cellular model to study lipid droplet function. Cells. (2019) 8:187. doi: 10.3390/cells8020187 30795549 PMC6406896

[17] Lefèvre C Jobard F Caux F Bouadjar B Karaduman A Heilig R. Mutations in CGI-58, the gene encoding a new protein of the esterase/lipase/thioesterase subfamily, in Chanarin-Dorfman syndrome. Am J Hum Genet. (2001) 69:1002–12. doi: 10.1086/324121 PMC1274347 11590543

[18] Heikinheimo P Goldman A Jeffries C Ollis DL. Of barn owls and bankers: a lush variety of alpha/beta hydrolases. Structure. (1999) 7:R141–6. doi: 10.1016/S0969-2126(99)80079-3 10404588

[19] Nardini M Dijkstra BW. Alpha/beta hydrolase fold enzymes: the family keeps growing. Curr Opin Struct Biol. (1999) 9:732–7. doi: 10.1016/S0959-440X(99)00037-8 10607665

[20] Lass A Zimmermann R Haemmerle G Riederer M Schoiswohl G Schweiger M. Adipose triglyceride lipase-mediated lipolysis of cellular fat stores is activated by CGI-58 and defective in Chanarin-Dorfman Syndrome. Cell Metab. (2006) 3:309–19. doi: 10.1016/j.cmet.2006.03.005 16679289

[21] Subramanian V Rothenberg A Gomez C Cohen AW Garcia A Bhattacharyya S. Perilipin A mediates the reversible binding of CGI-58 to lipid droplets in 3T3-L1 adipocytes. J Biol Chem. (2004) 279:42062–71. doi: 10.1074/jbc.M407462200 15292255

[22] Akiyama M Sakai K Takayama C Yanagi T Yamanaka Y McMillan JR. CGI-58 is an alpha/beta-hydrolase within lipid transporting lamellar granules of differentiated keratinocytes. Am J Pathol. (2008) 173:1349–60. doi: 10.2353/ajpath.2008.080005 PMC2570125 18832586

[23] Granneman JG Moore HP Krishnamoorthy R Rathod M. Perilipin controls lipolysis by regulating the interactions of AB-hydrolase containing 5 (Abhd5) and adipose triglyceride lipase (Atgl). J Biol Chem. (2009) 284:34538–44. doi: 10.1074/jbc.M109.068478 PMC2787315 19850935

[24] Yamaguchi T Omatsu N Matsushita S Osumi T. CGI-58 interacts with perilipin and is localized to lipid droplets. Possible involvement of CGI-58 mislocalization in Chanarin-Dorfman syndrome. J Biol Chem. (2004) 279:30490–7. doi: 10.1074/jbc.M403920200 15136565

[25] Sahu-Osen A Montero-Moran G Schittmayer M Fritz K Dinh A Chang YF. CGI-58/ABHD5 is phosphorylated on Ser239 by protein kinase A: control of subcellular localization. J Lipid Res. (2015) 56:109–21. doi: 10.1194/jlr.M055004 PMC4274058 25421061

[26] Granneman JG Moore HP Mottillo EP Zhu Z Zhou L. Interactions of perilipin-5 (Plin5) with adipose triglyceride lipase. J Biol Chem. (2011) 286:5126–35. doi: 10.1074/jbc.M110.180711 PMC3037624 21148142

[27] Brown JM Chung S Das A Shelness GS Rudel LL Yu L. CGI-58 facilitates the mobilization of cytoplasmic triglyceride for lipoprotein secretion in hepatoma cells. J Lipid Res. (2007) 48:2295–305. doi: 10.1194/jlr.M700279-JLR200 17664529

[28] Reina-Campos M Moscat J Diaz-Meco M. Metabolism shapes the tumor microenvironment. Curr Opin Cell Biol. (2017) 48:47–53. doi: 10.1016/j.ceb.2017.05.006 28605656 PMC5650101

[29] DeBerardinis RJ Mancuso A Daikhin E Nissim I Yudkoff M Wehrli S. Beyond aerobic glycolysis: transformed cells can engage in glutamine metabolism that exceeds the requirement for protein and nucleotide synthesis. Proc Natl Acad Sci U.S.A. (2007) 104:19345–50. doi: 10.1073/pnas.0709747104 PMC2148292 18032601

[30] Cheng C Geng F Li Z Zhong Y Wang H Cheng X. Ammonia stimulates SCAP/Insig dissociation and SREBP-1 activation to promote lipogenesis and tumour growth. Nat Metab. (2022) 4:575–88. doi: 10.1038/s42255-022-00568-y PMC9177652 35534729

[31] Paredes F Williams HC San Martin A. Metabolic adaptation in hypoxia and cancer. Cancer Lett. (2021) 502:133–42. doi: 10.1016/j.canlet.2020.12.020 PMC8158653 33444690

[32] Gu Y Chen Y Wei L Wu S Shen K Liu C. ABHD5 inhibits YAP-induced c-Met overexpression and colon cancer cell stemness via suppressing YAP methylation. Nat Commun. (2021) 12:6711. doi: 10.1038/s41467-021-26967-5 34795238 PMC8602706

[33] Ou J Miao H Ma Y Guo F Deng J Wei X. Loss of abhd5 promotes colorectal tumor development and progression by inducing aerobic glycolysis and epithelial-mesenchymal transition. Cell Rep. (2014) 9:1798–811. doi: 10.1016/j.celrep.2014.11.016 PMC4268306 25482557

[34] Ou J Peng Y Yang W Zhang Y Hao J Li F. ABHD5 blunts the sensitivity of colorectal cancer to fluorouracil via promoting autophagic uracil yield. Nat Commun. (2019) 10:1078. doi: 10.1038/s41467-019-08902-x 30842415 PMC6403256

[35] Peng Y Miao H Wu S Yang W Zhang Y Xie G. ABHD5 interacts with BECN1 to regulate autophagy and tumorigenesis of colon cancer independent of PNPLA2. Autophagy. (2016) 12:2167–82. doi: 10.1080/15548627.2016.1217380 PMC5103361 27559856

[36] Shang S Ji X Zhang L Chen J Li C Shi R. Macrophage ABHD5 suppresses NFκB-dependent matrix metalloproteinase expression and cancer metastasis. Cancer Res. (2019) 79:5513–26. doi: 10.1158/0008-5472.CAN-19-1059 31439546

[37] Miao H Ou J Peng Y Zhang X Chen Y Hao L. Macrophage ABHD5 promotes colorectal cancer growth by suppressing spermidine production by SRM. Nat Commun. (2016) 7:11716. doi: 10.1038/ncomms11716 27189574 PMC4873969

[38] Xu S Cai J Cheng H Wang W. Sustained release of therapeutic gene by injectable hydrogel for hepatocellular carcinoma. Int J Pharm X. (2023) 6:100195. doi: 10.1016/j.ijpx.2023.100195 37448985 PMC10336675

[39] Zhou Q Sun Y. Circular RNA cMras suppresses the progression of lung adenocarcinoma through ABHD5/ATGL axis using NF-κB signaling pathway. Cancer Biother Radiopharm. (2023) 38:336–46. doi: 10.1089/cbr.2020.3709 32822232

[40] Chen G Zhou G Aras S He Z Lucas S Podgorski I. Loss of ABHD5 promotes the aggressiveness of prostate cancer cells. Sci Rep. (2017) 7:13021. doi: 10.1038/s41598-017-13398-w 29026202 PMC5638841

[41] Chen G Zhou G Lotvola A Granneman JG Wang J. ABHD5 suppresses cancer cell anabolism through lipolysis-dependent activation of the AMPK/mTORC1 pathway. J Biol Chem. (2021) 296:100104. doi: 10.1074/jbc.RA120.014682 33219129 PMC7949079

[42] Mitra R Le TT Gorjala P Goodman OB Jr. Positive regulation of prostate cancer cell growth by lipid droplet forming and processing enzymes DGAT1 and ABHD5. BMC Cancer. (2017) 17:631. doi: 10.1186/s12885-017-3589-6 28877685 PMC5588693

[43] Zhou Q Wang F Zhou K Huang K Zhu Q Luo X. Oncogenic role of ABHD5 in endometrial cancer. Cancer Manag Res. (2019) 11:2139–50. doi: 10.2147/CMAR PMC6421882 30936746

[44] Shi Z Luo X Zhao H Huang B Wang Y Chen X. Clinicalpathologic and prognostic significance of CGI-58 in endometrial cancer. J Cancer. (2021) 12:7374–9. doi: 10.7150/jca.61905 PMC8734411 35003357

[45] Senchenko VN Kisseljova NP Ivanova TA Dmitriev AA Krasnov GS Kudryavtseva AV. Novel tumor suppressor candidates on chromosome 3 revealed by NotI-microarrays in cervical cancer. Epigenetics. (2013) 8:409–20. doi: 10.4161/epi.24233 PMC3674050 23478628

[46] Chen WS Chen PL Li J Lind AC Lu D. Lipid synthesis and processing proteins ABHD5, PGRMC1 and squalene synthase can serve as novel immunohistochemical markers for sebaceous neoplasms and differentiate sebaceous carcinoma from sebaceoma and basal cell carcinoma with clear cell features. J Cutan Pathol. (2013) 40:631–8. doi: 10.1111/cup.12147 23557589

[47] Plaza JA Mackinnon A Carrillo L Prieto VG Sangueza M Suster S. Role of immunohistochemistry in the diagnosis of sebaceous carcinoma: a clinicopathologic and immunohistochemical study. Am J Dermatopathol. (2015) 37:809–21. doi: 10.1097/DAD.0000000000000255 26485238

